# Analysis of the Electron Density of a Water Molecule Encapsulated by Two Cholic Acid Residues

**DOI:** 10.3390/ijms24065359

**Published:** 2023-03-10

**Authors:** María Pilar Vázquez-Tato, Julio A. Seijas, Francisco Meijide, Santiago de Frutos, José Vázquez Tato

**Affiliations:** 1 Departamento de Química Orgánica, Facultade de Ciencias, Universidade de Santiago de Compostela, Campus Terra, 27080 Lugo, Spain; 2 Departamento de Química Física, Facultade de Ciencias, Universidade de Santiago de Compostela, Campus Terra, 27080 Lugo, Spain

**Keywords:** bile acid, cholic acid, hydrogen bond, atoms in molecules theory, electronic density, critical points

## Abstract

Cholic acid is a trihydroxy bile acid with a nice peculiarity: the average distance between the oxygen atoms (O7 and O12) of the hydroxy groups located at C7 and C12 carbon atoms is 4.5 Å, a value which perfectly matches with the O/O tetrahedral edge distance in Ih ice. In the solid phase, they are involved in the formation of hydrogen bonds with other cholic acid units and solvents. This fact was satisfactorily used for designing a cholic dimer which encapsulates one single water molecule between two cholic residues, its oxygen atom (Ow) being exactly located at the centroid of a distorted tetrahedron formed by the four steroid hydroxy groups. The water molecule participates in four hydrogen bonds, with the water simultaneously being an acceptor from the 2 O12 (hydrogen lengths are 2.177 Å and 2.114 Å) and a donor towards the 2 O7 (hydrogen bond lengths are 1.866 Å and 1.920 Å). These facts suggest that this system can be a nice model for the theoretical study of the formation of ice-like structures. These are frequently proposed to describe the water structure found in a plethora of systems (water interfaces, metal complexes, solubilized hydrophobic species, proteins, and confined carbon nanotubes). The above tetrahedral structure is proposed as a reference model for those systems, and the results obtained from the application of the atoms in molecules theory are presented here. Furthermore, the structure of the whole system allows a division into two interesting subsystems in which water is the acceptor of one hydrogen bond and the donor of another. The analysis of the calculated electron density is performed through its gradient vector and the Laplacian. The calculation of the complexation energy used correction of the basis set superposition error (BSSE) with the counterpoise method. As expected, four critical points located in the H…O bond paths were identified. All calculated parameters obey the proposed criteria for hydrogen bonds. The total energy for the interaction in the tetrahedral structure is 54.29 kJ/mol, while the summation obtained of the two independent subsystems and the one between the alkyl rings without water is only 2.5 kJ/mol higher. This concordance, together with the calculated values for the electron density, the Laplacian of the electron density, and the lengths of the oxygen atom and the hydrogen atom (involved in the formation of each hydrogen bond) to the hydrogen bond critical point, suggests that each pair of hydrogen bonds can be considered independent of each other.

## 1. Introduction

During evolution, nature has learned to distinguish what works from what does not. Consequently, all living beings have adopted successful mechanisms and molecules for solving the challenges they must face to achieve a particular purpose. The knowledge of the involved processes provides the scientific community with strategies for designing new molecules with specific properties to reach a desired target. According to Menger [1], the design of a molecule from the beginning with a list of optimal functionalities is not an easy task, although it may be facilitated by emulating the biological mechanisms. Among many other molecules, bile acids fulfill the steps described by Lenh [2] involved in the evolution of matter. Hofmann [3] has discussed their structural variation and its possible evolutionary significance. The steroid nucleus has been implied in a key evolutionary step as it is ubiquitous in animals (as hormones, cholesterol, and bile acids) and plants (as brassinosteroids).

All the above information led us to use natural bile acids as raw materials for synthesizing derivatives that self-organize into new supramolecular structures [4,5,6,7]. Among the designs, we studied a cholic dimer, which encapsulates one single water molecule between two cholic residues [8].

**Figure 1:**
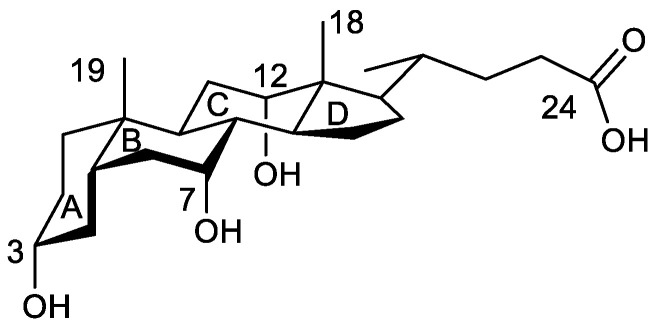
Structure of cholic acid. Significant carbon atoms are numbered as well as the four rings of the steroid nucleus. In the text, the numbers of oxygen atoms are those of the carbon atoms to which they are bonded.

**Figure 2:**
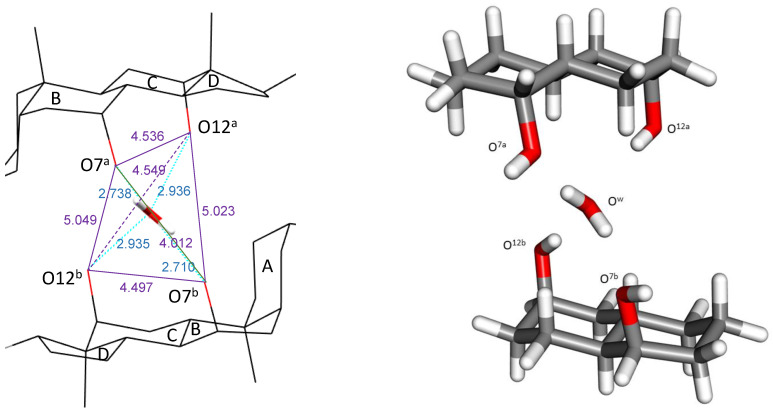
**Left**: Oxygen–Oxygen distances (lines and data in amethyst color) of the tetrahedron formed by the O^7^-H and O^12^-H hydroxy atoms of the two steroid residues encapsulating a water molecule located at their centroid [8]. The four hydrogen bonds are indicated with blue lines, as are the hydrogen bond distance values (O^w^-H^…^O^7^ and O^12^-H^…^O^w^). All data in Å. **Right**: Simplified system model.

Bile acids (BAs) have a bifacial polarity since the hydroxy groups (up to three at C3, C7, and C12 carbon atoms) lie beneath the plane of the steroid nucleus (hydrophilic α-side). The characterization of the crystal structures of BA and their derivatives by X-ray analysis has been a topic of interest for years [9,10,11,12,13,14,15,16,17]. Common to all crystal structures is that the hydroxy groups are always involved in the formation of hydrogen bonds (HB) with other BA molecules, the solvent, or both species. In cholic acid (Figure 1), the average hydrogen bond distances formed by the C7-OH and C12-OH hydroxy groups (from here these oxygen atoms will be identified as O^7^ and O^12^) with water have been recompiled with values of 2.79 ± 0.09 Å and 2.86 ± 0.10 Å, respectively [9], the O^7^/O^12^ distance being 4.5 Å, a value which perfectly matches with the O/O tetrahedral edge distance in I_h_ ice, respectively [18,19]. These facts suggest the design of the cholic dimer mentioned previously [8]. In this complex, the water oxygen atom (O^w^) is exactly located at the centroid of a distorted tetrahedron formed by the four steroid hydroxy groups (Figure 2, left). Both O^12^-H are hydrogen bond donors towards O^w^, while both O^7^-H are acceptors from O^w^. Figure 2 (right) shows the values for the four hydrogen bonds. It may be noticed that the O^7…^O^w^ distances are close to the one measured in I_h_ ice while the O^12…^O^w^ distances match the O^…^O distance observed in the water dimer in gas phase (see below). In his review on the hydrogen bond in the solid state, Steiner [20] has indicated average values of 1.880(2) Å and 2.825(2) Å for H^…^OH_2_ and O^…^O distances, respectively, for the dimer HO-H^…^OH_2_.

The term ice-like is frequently used to describe the structure of water found in a plethora of systems. Among them, we can mention water clusters (H_2_O)_n_ in compounds as metal-organic networks in the solid state [21], liquid water solubilizing hydrophobic species [22,23,24,25] or proteins [26,27], and in water interfaces [28]. However, Bonn et al. [29] have concluded that the vibrational spectrum of water at both water-lipid and water-protein interfaces is inconsistent with the presence of “ice-like” structures. Ice-like behavior is also recognized in carbon nanotubes (CNTs) [30,31,32,33] and in sub-nanometer carbon slit pores [34], but it can be suppressed in supercooled water in tight confinements [35].

Weissmann et al. [36] self-limited their study on the hydrogen bond in an ice-like structure to “the interactions of one water molecule with its four nearest neighbors” somehow accepting that a water molecule should form four hydrogen bonds, the oxygen atom simultaneously being a hydrogen bond donor and acceptor (two of each). Therefore, in the analysis of published structures that we have carried out, only tetrahedral water and the interaction with neutral oxygen atoms have been considered. Different O^…^O hydrogen bond distances are observed in water clusters in metal-organic complexes, depending on the role of the oxygen atom as acceptor or donor of a hydrogen bond [21,37,38]. This difference can be as high as 0.17 Å (measured from cif files) [38]. In our opinion, this distinction has not been sufficiently analyzed in the literature. Obviously, such a distinction cannot be made in I_h_ ice, as all O^…^O hydrogen bond distances have the same value.

All the previous facts, together with the perfect distinction between hydrogen bond donors and acceptors for water linked to a tetrahedral hydroxy structure, surrounded by apolar alkyl skeletons, constitute a unique model to pursue a theoretical study. Keeping this in mind, the “atoms in molecules” (AIM) theory [39,40] has been applied to a model system derived from this ice-like structure. On the other hand, when analyzing BA crystals for the acceptance of the formation of a hydrogen bond, the geometric criteria (bond lengths and angles) [41] have been used exclusively. This is the first time that the AIM theory has been applied to a BA crystal.

## 2. Results and Discussion

### 2.1. Complex O^12a^-H^…^O^w^-H^…^O^7a^//O^12b^-H^…^O^w^-H^…^O^7b^

**Figure 3:**
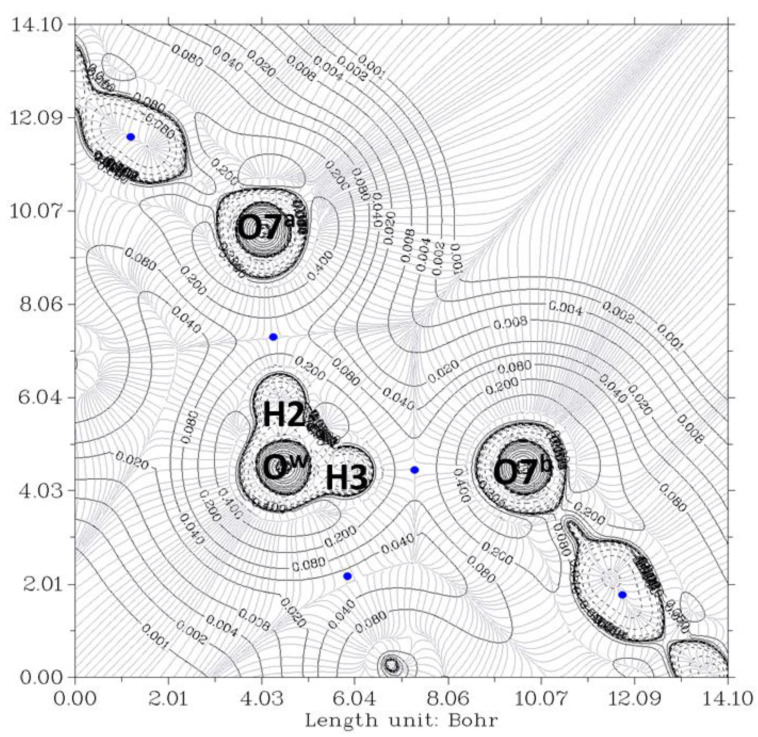
The electron density contour of the O^12a^-H^…^O^w^-H^…^O^7a^//O^12b^-H^…^O^w^-H^…^O^7b^ complex (thin black lines) and BCP (3,-1) (blue dots). The plane is defined by O^7a^, O^7b^, and O^w^ oxygen atoms of the pseudo-bile acid residues and water, respectively. Thin gray lines correspond to the gradient of the electron density.

**Figure 4:**
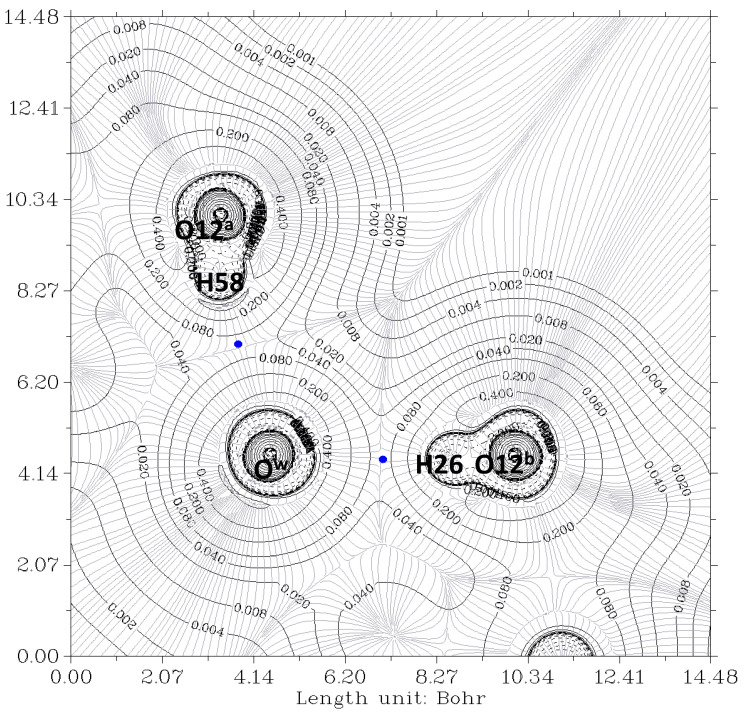
The electron density contour of the O^12a^-H^…^O^w^-H^…^O^7a^//O^12b^-H^…^O^w^-H^…^O^7b^ complex (thin black lines) and BCP (3,-1) (blue dots). The plane is defined by the O^12a^, O^12b^, and O^w^ oxygen atoms of the bile acid residues and water, respectively. Thin gray lines correspond to the gradient of the electron density.

The electron density, ρ, is the starting point of the AIM theory. Its topology is easily deduced from the gradient vector, ∇ρ, and the Laplacian, ∇^2^ρ. The electron density is usually visualized by drawing contour lines connecting electron density points with the same value. Figure 3 and Figure 4 show two examples for the present system. In Figure 3, the plane is defined by the oxygen nuclei O^7a^, O^7b^, and O^w^, while in Figure 4, the plane is defined by oxygen atoms O^12a^, O^12b^, and O^w^. The thin gray lines are defined by infinitesimal gradient vectors, which describe gradient paths. 

**Figure 5:**
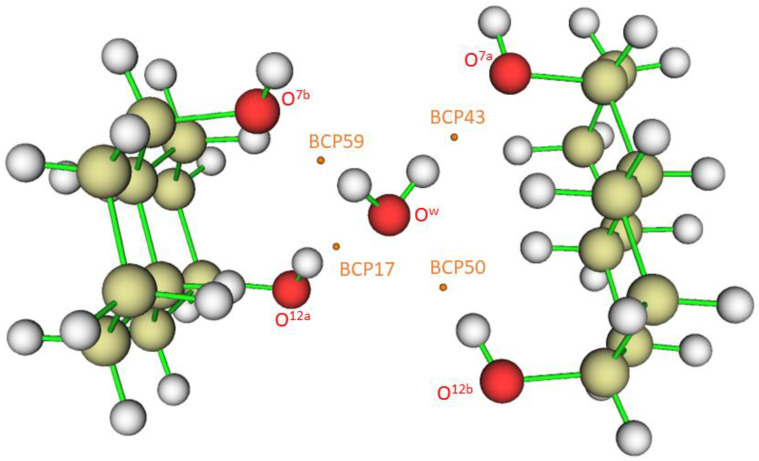
Bond critical points (BCP) and hydrogen bond critical points (HBCP) obtained for the complex O^12a^-H^…^O^w^-H^…^O^7a^//O^12b^-H^…^O^w^-H^…^O^7b^. HBCPs are identified with numbers 17, 43, 50, and 59.

When ∇^2^ρ < 0, the electronic charge is locally concentrated, as in the case of covalent bonds [42]. When ∇^2^ρ > 0, the electronic charge is locally depleted [40], resulting in what are called *closed-shell* interactions. This happens in hydrogen bonds (HB), in which the charge concentrations are separately localized in the basins of the neighboring atoms [43]. Figure 5 shows bond critical points where the gradient ∇ρ vanishes. Numbers 17, 43, 50, and 59, located between hydrogen and oxygen atoms, correspond to hydrogen bond critical points (HBCP), which are (3,−1) saddle points. Other numbers correspond to covalent bonds (located, for instance, between two carbon atoms).

In Figure 3, the contour lines of the electronic density around the water oxygen (O^w^) basin resemble a Mickey Mouse profile. This is a consequence of the fact that the two hydrogen atoms of water (named H2 and H3 in the Figure) form covalent bonds with O^w^. In other words, O^w^ is behaving as a HB donor, while from this perspective, the basin of the O^7^ oxygen atom has a circle shape. Similarly, Figure 4 shows the contour lines of the hydrogen bonds between O^w^ and the two O^12^-H hydroxy groups. The plane in the Figure is defined by these three oxygen atoms. Now the contour around the O^w^ resembles a basin, while the profiles around the O^12^-H groups resemble peanuts. The two O^12^ are donors, and O^w^ is the acceptor. Furthermore, the bond paths of the four hydrogen bonds link the expected two atoms, the hydrogen and the acceptor. It is evident that the first condition of the criteria to characterize a hydrogen bond published by Popelier [42,44] is fulfilled. Furthermore, according to Popelier [42], the ρ values at the HBCP, ρ_b_, should be in the range 0.002−0.035 au, Table 1 showing that this is the case for the four HB. These values are about one order of magnitude smaller than those found for a covalent bond (ρ_b_ = 0.391 au, for O-H in H_2_O) [45]. On the other hand, it may be noticed that the values when water is a donor (towards O^7^) are almost double than when it behaves as an acceptor (from O^12^). 

The correlation between the O–O length and ρ_b_ has been published [46,47]. The shorter the former, the higher the latter. The values obtained here differ by less than ±0.004 au with those obtained from the equation $\rho_{b}=2.71\times exp\left(-2.40\times r_{O\ldots H}\right)$, ($r_{O\ldots H}$ in Å) [47]. They also match values recompiled by Steiner [20] (see Figure 3 of this reference).

A third criterion proposed by Popelier refers to the Laplacian of the charge density evaluated at the bond critical point, where charge density is a local minimum along the bond path, i.e., ρ_b_ is locally depleted with respect to neighboring points along the bond path. The range values (Table 1) are also within the proposed range of 0.024–0.139 au. ∇^2^ρ_b_ follows an analogous dependence with the O-O length than ρ_b_.

Previous ρ_b_ and ∇^2^ρ_b_ values may be compared with those for the water dimer, H-O-H^…^OH_2_ in the gas phase. The water dimer is a system of two water molecules bound by a single hydrogen bond, often used as the paradigmatic system [48,49]. Its equilibrium geometry is well-known, as is the dissociation energy. The dimer has a “trans-linear” structure, and the O^…^O distance was first measured by Dyke et al. from the microwave spectrum [50,51,52], the value being *r_O…O_* = 2.98 ± 0.04 Å. Lane [53] has calculated a value of *r_O…O_* = 2.91 Å (truncated value to the hundredth of Å) as the best estimation. The O-H distances depend on the role of the water molecules, with values of *r_OH_* = 0.958 pm Å and *r_OH_* = 0.95 Å when water is a donor or acceptor, respectively [48]. Bader et al. [45] have obtained that ρ_b_ and ∇^2^ρ_b_ are 0.0199 and 0.0624 (data in au), respectively. Other values can be found elsewhere [54,55]. From the four values of ρ_b_ (Table 1), an average value of 0.020 au is obtained, which matches the one for the water dimer. The ∇^2^ρ_b_ value for H-O-H^…^OH_2_ is closer to those in which the oxygens (O^12^) of hydroxy groups are donors and O^w^ is an acceptor. It should be noticed that in these last two cases, the *r_OO_* lengths are also closer to that of the H-O-H^…^OH_2_ dimer. 

**Figure 6:**
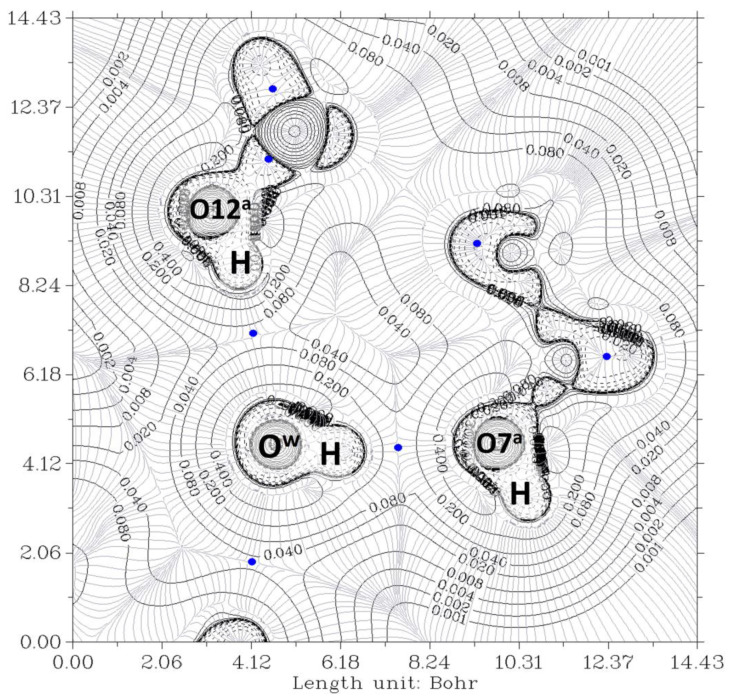
The electron density contour of the O^12a^-H^…^O^w^-H^…^O^7a^ complex (thin black lines) and BCP (3,-1) (blue dots). O^12a^, O^7a^, and O^w^ are oxygen atoms from the pseudo-bile acid residues and water, respectively. Thin gray lines correspond to the gradient of the electron density.

The mutual penetration of the hydrogen (H) and acceptor atoms (A, oxygen) is another criterion of hydrogen bond formation. This criterion is often considered a necessary and sufficient condition for the classification of an intermolecular interaction as hydrogen bonding [56]. It is estimated as $∆r_{i}=r_{i}-r_{i}^{o}$ (*i*, are the atoms involved in the hydrogen bond, A or H), *r_i_* being the bonded radius of each atom and $r_{i}^{o}$ the corresponding nonbonded radius [44]. The nonbonded radius is the distance of a nucleus from a given electron density contour (usually 0.001 au) in the absence of interaction. This value is taken because it yields atomic diameters in good agreement with van der Waals radii in the gas phase [44]. The bonded radius is the distance from a nucleus to the bond critical point (HBCP). Numbers 17, 43, 50, and 59 identify these HBCPs in Figure 6. Table 1 shows the HBCP^…^O and HBCP^…^H lengths calculated for the complex. Obviously, the sum of both lengths should coincide with the imposed one from the crystal (*r*_1_+ *r*_2_ = *r*_O_…_H_ length in Table 1). The HBCP^…^H length, *r*_2_, for the hydrogen bonds with O^w^ as acceptor is larger (>0.1 Å) than those for O^w^ being the donor. All of them are considerable smaller than this distance for the water dimer in the gas phase (=1.34 Å). Accepting that $r_{H}^{o}=r_{vdW}^{H}$ = 1.1 Å [57], $∆r_{H}\;$< 0 in all cases. Similarly, if $r_{vdW}^{O}=r_{O}^{o}$ = 1.58 Å [57], then $∆r_{O}\;$< 0. These data provide evidence of a mutual penetration of hydrogen and oxygen atoms, a conclusion which may be raised from checking the contour electron density values of Figure 4 and Figure 5. It should be noted that Isaev has defined [56] $∆r_{i}=r_{i}^{o}-r_{i}$, i.e., $∆r_{O}=r_{vdW}^{o}-r_{O\ldots HBCP}$ and $∆r_{H}=r_{vdW}^{H}-r_{H\ldots HBCP}$. In all cases, $∆r_{H}>∆r_{O}$, meaning that the hydrogen atom is more penetrated than the acceptor one.

### 2.2. Complexes O^12a^-H^…^O^w^-H^…^O^7a^, O^12b^-H^…^O^w^-H^…^O^7b^ and O^12a^-H/H^…^O^7a^//O^12b^-H/H^…^O^7b^

Without changing the coordinates of the atoms, the whole complex O^12a^-H^…^O^w^-H^…^O^7a^//O^12b^-H^…^O^w^-H^…^O^7b^ can be divided into two independent complexes, O^12a^-H^…^O^w^-H^…^O^7a^ and O^12b^-H^…^O^w^-H^…^O^7b^, in which the water molecule only interacts with one of the pseudo-steroid residues of the original complex. It must be noticed that in both hemicomplexes, the water molecule is participating in the formation of two hydrogen bonds, being an acceptor and donor towards the O^12^ and O^7^ oxygen atoms, respectively. Thus, in each complex, only one O-H bond of water participates as a donor.

**Figure 7:**
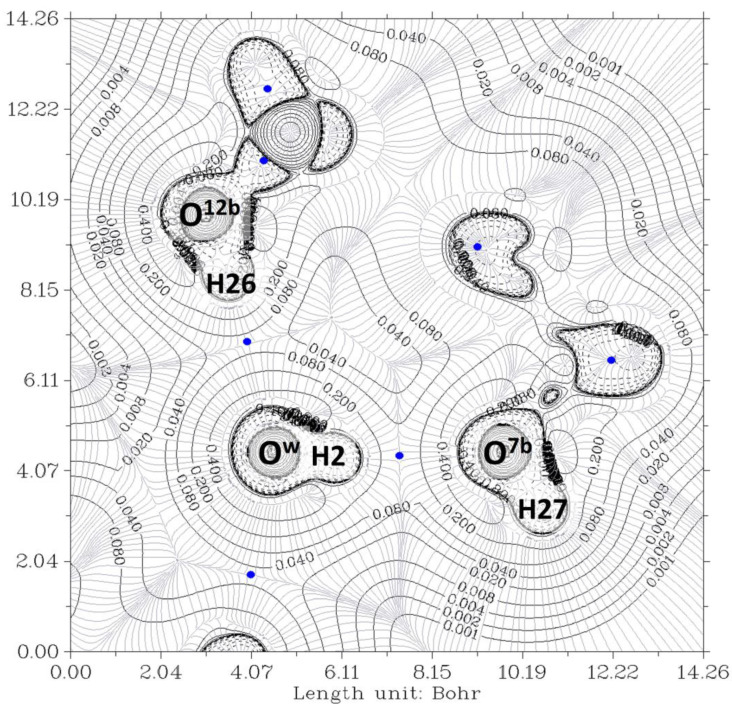
The electron density contour of the O^7b^-O^w^-O^12b^ complex (thin black lines) and BCP (3,-1) (blue dots). O^12b^, O^7b^, and O^w^ are oxygen atoms from the pseudo-bile acid residues and water, respectively. Thin gray lines correspond to the gradient of the electron density.

Figure 6 and Figure 7 show the contour lines at the planes defined by O^7a^-O^w^-O^12a^ and O^7b^-O^w^-O^12b^, respectively. In both cases, O^w^ and O^12^ exhibit peanut profiles directed towards the associated acceptor atoms O^7^ and O^w^, respectively. In all cases, the bond paths of the four hydrogen bonds link the expected two atoms, and the HBCP is indicated with a blue color point. The analysis of the data was carried out as previously. The HBCPs are identified by numbers (Figure not shown), and, for association purposes, the HBCPs of the full complex are shown in brackets. Table 2 shows the obtained results.

It may be observed that all the values in Table 2 perfectly match those in Table 1. This is partially due to the fact that the original geometric parameters of the C-H_2_O-C crystal are kept constant. Because of previous agreements, the mutual penetration of hydrogen and oxygen atoms is not discussed. 

**Figure 8:**
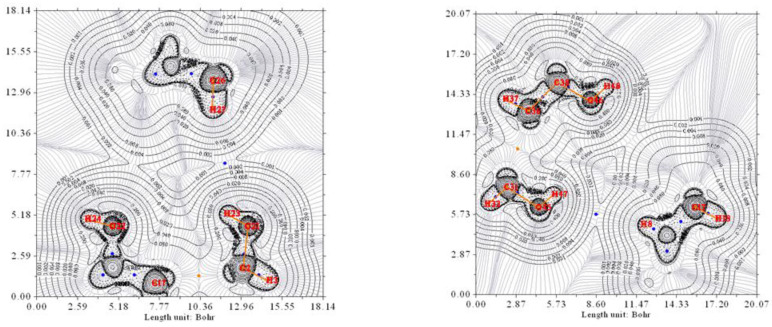
The electron density contour of the O^12a^-H/H^…^O^7a^//O^12b^-H/H^…^O^7b^ complex (thin black lines). O^7a^ and O^7b^ are oxygen atoms from the pseudo-bile acid residues. Thin gray lines correspond to the gradient of the electron density.

Finally, the electron density of the complex without water, O^12a^-H/H^…^O^7a^//O^12b^-H/H^…^O^7b^, has also been studied. Figure 8 shows the contour map of the two halves of the complex. As it was expected, the contour lines strongly differ from previous ones, and HBCP are not observed. 

### 2.3. Energy of Hydrogen Bonds

The interaction energy for the formation of the complex O^12a^-H^…^O^w^-H^…^O^7a^//O^12b^-H^…^O^w^-H^…^O^7b^ is −54.29 kJ mol^−1^. This value cannot be exclusively ascribed to the formation of the four hydrogen bonds. In fact, for the complex O^12a^-H/H^…^O^7a^//O^12b^-H/H^…^O^7b^ (in which the water molecule has been removed), a value of −4.60 kJ/mol has been calculated. Because of the length differences between the hydrogen bonds in which O^w^ is the donor and those in which it is the acceptor (2.7 Å vs. 2.9 Å), the energy of each hydrogen bond is expected to be different [58]. Having these considerations in mind, the average energy of the hydrogen bonds is −13.57 kJ/mol. Such a value indicates that they are moderate hydrogen bonds according to Jeffrey’s categories [59] or weak to medium according to the ranges proposed by Emamian et al. [60]. Rocher-Casterline et al. [61] have determined a value of 13.2 ± 0.5 kJ mol^−1^ for the bond dissociation energy (*D*_o_) of the water dimer. Ruscic [62], from a new partition function for water, has obtained dissociation enthalpy values for the water dimer, the values being 13.220 ± 0.096 kJ mol^−1^ and 15.454 ± 0.074 kJ mol^−1^ at 0 K and 298.15 K, respectively, and Feyereisen et al. [63], from the thermal conductivity of the vapor, measured a value of −15.07 ± 2.1 kJ mol^−1^. Most of the values calculated theoretically for this dimer are within the interval −13.4/−23.1 kJ mol^−1^ [45,53,54,55,60,63,64,65,66]. 

For the formation of hydrogen bonds between water and methanol, in gas phase, Moin et al. [67] have obtained values of 1.96–2.04 Å (O_meth_H^…^O_w_) and 1.94–2.02 Å (O_w_H^…^O_meth_), for the H^…^O distances, while the hydrogen bond energies were in the ranges of −20.45/−27.04 kJ mol^−1^ (O_meth_H^…^O_w_) and −21.24/−29.39 kJ mol^−1^ (O_w_H^…^O_meth_), the values depending on the level of the theory. These values are in line with the different behavior of water depending on whether it is a donor or acceptor, as observed previously.

For a series of hydrogen-bonded complexes between nitrites and hydrogen chloride, Boyd and Choi [68] have noticed a correlation between the electron density at the HBCP ρ_b_ and the energy of the hydrogen bond. The energies ranged from 10 kJ/mol to 38 kJmol, while the range of ρ_b_ was 0.01103−0.02391. Many other equations have been published; the subject is being reviewed by Rozenberg [69]. There is no objective reason to choose one or another equation for the present system, and as an orientation, we will use the following relationship that Rozenberg obtained from 24 equations:

E(kJ/mol) = −(6.6 ± 8.0) + (1215 ± 440)ρ

After its application to each hydrogen bond of the present system, the summation of the individual values gives a total energy of −72 ± 24 kJ/mol, with a high standard deviation. 

As indicated above, the nature of the complex O^12a^-H/H^…^O^7a^//O^12b^-H/H^…^O^7b^ allows the calculation of the interaction of two subsystems, O^12a^-H^…^O^w^-H^…^O^7a^ and O^12b^-H^…^O^w^-H^…^O^7b^, both having two hydrogen bonds with water acting as donor and acceptor. The calculated values are −25.91 kJ/mol and −21.18 kJ/mol for the “a” and “b” subsystems, respectively. By considering the interaction energy between the two pseudosteroids) (see above) and the previous values, the difference between the calculated energy of the whole system and that resulting from the sum of the three subsystems is only 2.5 kJ/mol. 

## 3. Materials and Methods

### Crystal Structure and Computational Details

The crystal structure of the reference system (C-H_2_O-C) was previously published [8]. In this reference, a complete image of Figure 2, is shown on the left. The Cif files (CCDC 867499) contain the supplementary crystallographic data for the C-suc-C crystal (the acronym given in that paper). These data can be obtained free of charge from the Cambridge Crystallographic Data Center via www.ccdc.cam.ac.uk/data_request/cif.

Given the high number of atoms involved in the two bile acid dimers, to analyze the interaction with the water molecule, we have simplified the system by reducing the number of atoms in the bile acid unit while keeping the same geometric parameters of the remaining atoms. Thus, A and D rings were suppressed, and the carbon atoms linking them to B and C rings were replaced by hydrogen atoms. This system will be referred to as O^12a^-H^…^O^w^-H^…^O^7a^//O^12b^-H^…^O^w^-H^…^O^7b^, where the superscripts “a” and “b” refer to the upper and lower pseudo-bile acid residues, respectively (see Figure 2). This complex is later divided into two independent subsystems, named O^12a^-H^…^O^w^-H^…^O^7a^ and O^12b^-H^…^O^w^-H, which allow the calculation of the interaction of the water molecule with only one of the pseudo-steroid residues. The interaction between the two pseudo-bile acid residues, without water complexed between them, O^12a^-H/H^…^O^7a^//O^12b^-H/H^…^O^7b^, has also been studied.

We have maintained the original interatomic distances obtained from the x-ray resolution of the C-H_2_O-C complex, and no minimization of the energy of the complex was carried out. Calculations of the complexation energy used for correction of the basis set superposition error (BSSE) with the counterpoise method implemented in Gaussian 19 [70]. Laplacian of electronic density and critical points (AIM) were calculated using the Multiwfn_3.8_dev software [71].

## 4. Conclusions

There are two main oxygen-oxygen (*r_OO_*) distances when a hydrogen bond is formed between water molecules: the one observed in the gas phase in the formation of a dimer (*r_OO_* = 2.98 Å) and the one in ice (*r_OO_* = 2.75 Å). Both lengths are observed in the C-succ-C crystal, in which a water molecule is encapsulated by four hydroxy groups belonging to two cholic acid dimers. The shorter one corresponds to hydrogen bonds in which the water oxygen is donor and the larger one when it is the acceptor. The application of the AIM theory to a simplified system O^12a^-H^…^O^w^-H^…^O^7a^//O^12b^-H^…^O^w^-H^…^O^7b^ confirms the existence of saddle critical points (HBCP) in all four of these hydrogen bonds. The estimated interaction energy in the formation of the complex, −54.29 kJ mol^−1^, is in acceptable agreement with the summation of the energies of the two hemicomplexes, O^12a^-H^…^O^w^-H^…^O^7a^ and O^12b^-H^…^O^w^-H^…^O^7b^, in which the water molecule forms two hydrogen bonds (acting as donor and acceptor) and the interaction energy of the two pseudo-steroid nucleus O^12a^-H/H^…^O^7a^//O^12b^-H/H^…^O^7b^ (i.e., without complexed water). This fact and the calculated values for the electron density, the Laplacian of the electron density, and the lengths of the oxygen atom and the hydrogen atom (involved in the formation of each hydrogen bond) to the HBCP suggest that each pair of hydrogen bonds can be considered independent of each other.

## Figures and Tables

**Table 1:** Lengths involved in the formation of hydrogen bonds were determined from the crystal structure and calculated along with electron density and Laplacian values.

	Complex O^12a^-H^…^O^w^-H^…^O^7a^//O^12b^-H^…^O^w^-H^…^O^7b^			
Identification HBCP (Figure 5)	50	59	17	43
Property at HBCP	O^12a^-H^…^O^w^	O^w^-H^…^O^7a^	O^12b^-H^…^O^w^	O^w^-H^…^O^7b^
O-O length/Å crystal	2.936	2.738	2.935	2.710
O^…^H length/Å crystal	2.114	1.920	2.177	1.866
Electron density ρ_b,_ au	0.0154	0.0239	0.0138	0.0270
ρ_b_ calculated according to [47]	0.0170	0.0270	0.0146	0.0308
Laplacian of the electron density at HBCP, ∇^2^ρ_b,_ au	0.0667	0.106	0.0616	0.118
HBCP…O length/ Å, *r*_1_	1.355	1.242	1.383	1.217
HBCP…H length/ Å, *r*_2_	0.759	0.679	0.795	0.650
*r*_1_+ *r*_2_ = *r*_O_…_H_ length/Å	2.114	1.921	2.178	1.867
$∆r_{O}=r_{vdW}^{o}-r_{1}$/Å	0.225	0.338	0.197	0.363
$∆r_{H}=r_{vdW}^{H}-r_{2}/Å$	0.341	0.421	0.305	0.450

**Table 2:** Electron density, Laplacian of the electron density, and lengths involved in the formation of hydrogen bonds of the two semi complexes: O^12a^-H^…^O^w^-H^…^O^7a^ and O^12a^-H^…^O^w^-H^…^O^7a^.

	O^12a^-H^…^O^w^-H^…^O^7a^		O^12b^-H^…^O^w^-H^…^O^7b^	
Property at HBCP	CP71 (CP50) O^12a^-H^…^O^w^	CP74 (CP59) O^w^-H^…^O^7a^	CP68 (CP17) O^12b^-H^…^O^w^	CP71 (CP43) O^w^-H^…O7b^
Electron density ρ_b,_ au	0.0152	0.0239	0.0137	0.0270
Laplacian of ρ_b_, ∇^2^ρ_b,_ au	0.0662	0.106	0.0610	0.119
HBCP…O length/Å, *r*_1_	1.353	1.243	1.381	1.218
HBCP…H length/Å, *r*_2_	0.761	0.679	0.797	0.650
*r*_1_+ *r*_2_ = O…H length/Å	2.115	1.922	2.177	1.867
